# Surveillance-Associated Anxiety After Curative-Intent Cancer Surgery: A Systematic Review

**DOI:** 10.1245/s10434-024-16287-5

**Published:** 2024-09-29

**Authors:** Rakhsha Khatri, Patrick L. Quinn, Sharla Wells-Di Gregorio, Timothy M. Pawlik, Jordan M. Cloyd

**Affiliations:** 1 Division of Surgical Oncology, Department of Surgery, The Ohio State University Wexner Medical Center, Columbus, OH USA; 2 Division of Palliative Medicine, Department of Internal Medicine, The Ohio State University Wexner Medical Center, Columbus, OH USA

**Keywords:** Scanxiety, Anxiety, Fear of recurrence, Imaging, Follow-up care, Psycho-oncology, Cancer surgery

## Abstract

**Background:**

Regular surveillance imaging is commonly used after curative-intent resection of most solid-organ cancers to enable prompt diagnosis and management of recurrent disease. Given the fear of cancer recurrence, surveillance may lead to distress and anxiety (“scanxiety”) but its frequency, severity, and management among cancer survivors are poorly understood.

**Methods:**

A systematic review of the PubMed, Embase, CINAHL, and PsycINFO databases was conducted to evaluate existing literature on anxiety and emotional experiences associated with surveillance after curative-intent cancer surgery as well as interventions aimed at reducing scanxiety.

**Results:**

Across the 22 included studies encompassing 8693 patients, reported rates of scanxiety varied significantly, but tended to decrease as time elapsed after surgery. Qualitative studies showed that scanxiety arises from various factors innate to the surveillance experience and is most prevalent in the scan-to-results waiting period. Common risk factors for scanxiety included sociodemographic and cancer-related characteristics, low coping self-efficacy, pre-existing anxiety, and low patient well-being. Conversely, reassurance was a positive aspect of surveillance reported in several studies. Trials evaluating the impact of interventions all focused on modifying the surveillance regimen compared with usual care, but none led to reduced rates of scanxiety.

**Conclusions:**

Although scanxiety is nearly universal across multiple cancer types and patient populations, it is transient and generally limited in severity. Because existing trials evaluating interventions to reduce scanxiety have not identified effective strategies to date, future research is needed to identify interventions aimed at reducing their impact on high-risk individuals.

**Supplementary Information:**

The online version contains supplementary material available at 10.1245/s10434-024-16287-5.

Radiographic testing is essential to the diagnosis, treatment, and follow-up evaluation of patients with cancer. After multimodal treatment, cancer survivors continue to undergo scans and follow-up care regularly to monitor for cancer recurrence.^1^ Given its consequential nature, surveillance imaging can provoke anxiety and distress in many cancer survivors. This surveillance-associated anxiety, more commonly known as “scanxiety”, encompasses symptoms associated with any component of surveillance care, such as imaging, labs, or clinical assessment.^2,3^

Multiple studies have demonstrated that scanxiety is complex and multidimensional, with symptoms extending from the period leading up to the surveillance encounter until results are discussed and beyond.^4–6^ These experiences can have a significant impact on patients’ well-being and quality of life.^7^

Surgical resection is a necessary component for most solid-organ cancers. After resection, patients typically require at least 5 years of dedicated cancer surveillance, but many individuals will have lifelong follow-up evaluation. A key source of scanxiety is the fear of cancer recurrence (FCR), but other factors include discomfort from the exam, stress around scheduling, reliving the initial cancer diagnosis, and the costs incurred from such exams.^8^ With each surveillance encounter, patients may experience cognitive, behavioral, emotional, or even physical distress.

Previous studies and systematic reviews have focused on the prevalence of scanxiety and factors associated with it, primarily among patients undergoing cancer screening, active cancer surveillance, or assessment of treatment response. Given the unique experiences and implications of surveillance among postoperative cancer survivors, this systematic review aimed to characterize scanxiety among patients who had previously undergone curative-intent cancer surgery. Specifically, we sought to critically evaluate the existing literature on anxiety and emotional experiences associated with postoperative surveillance as well as interventions aimed at reducing surveillance-associated anxiety. An improved understanding of surveillance-associated distress after curative-intent cancer surgery might inform patient-centered interventions focused on enhancing quality of life.

## Methods

### Search Strategy and Study Selection

A systematic review of the PubMed, Embase, CINAHL, and PsycINFO databases was performed to evaluate the existing literature on psychological experiences associated with surveillance after curative-intent cancer surgery, as well as interventions patients may have received to mitigate surveillance-associated anxiety. The following concepts and search terms were formulated to be covered by the search strategy: emotional experience (e.g., “anxiety”, “scanxiety”, “nervous”, “anxious”, “psychological distress”, “emotional stress”, “fear”, “worry”, “apprehension”), surveillance (e.g., “surveillance,” “monitor,” “follow-up”), cancer (e.g., “neoplasm,” “carcinoma”, “tumor”), and surgery (e.g., “surgical”, “operative”, “resection”). The strategy considered both free-text terms and controlled vocabulary (e.g., MeSH and Emtree) to ensure a comprehensive search (Table S1).

This review included only English language studies with human subjects. The search was not limited to any specific type of malignancy. However, only studies of patients under surveillance after curative-intent cancer surgery (i.e., solid-organ cancers) were included. Studies could include any type of surveillance, including imaging, labs, or clinical exams. We excluded literature in which scanxiety was associated with the surveillance of metastatic cancer, diagnostic screening, or active surveillance. The review excluded published reports with non-primary source data, reviews, commentaries, letters, narratives, small case series (*n* < 10), case reports, and conference abstracts. Two reviewers independently screened studies by title, abstract, and full text using Covidence (Melbourne, Australia). All searches were completed in January 2024.

### Data Extraction

Data extraction from the included studies was undertaken by two authors independently, with disagreements settled by a third reviewer. The patient information abstracted included age, sex, cancer type and stage, curative-intent procedure, time elapsed since surgery, adjuvant therapy, and surveillance type. The study information abstracted included publication year, country of origin, number of participants, assessment tools and time points, comparison groups, and intervention characteristics.

### Statistical Analysis

Summary statistics are reported as total and percentage for categorical variables and as median values and interquartile ranges, unless stated otherwise, for continuous variables. The results were not pooled into a meta-analysis due to the variation and substantial heterogeneity among the included studies. The results of this systematic review are presented according to the PRISMA-P checklist (Table S6).^12^

### Quality Appraisal

To assess study quality and the presence of biases, each study underwent quality appraisal using the Joanna Briggs Institute (JBI) Critical Appraisal checklists (Tables S2–S5).The JBI tool for randomized controlled trials is composed of 13 questions regarding the study design, with the option to answer “yes” (indicating high quality), “no” (indicating poor quality), “unclear”, or “not applicable”.^9^ Similarly, the JBI checklist for cross-sectional, cohort/case-control, and qualitative studies consisted of 8, 11, and 10 questions, respectively.^10,11^ To prevent missing potentially valuable data, no studies were omitted based on this methodologic critical appraisal.

## Results

### Overall Study Characteristics

**Fig. 1:**
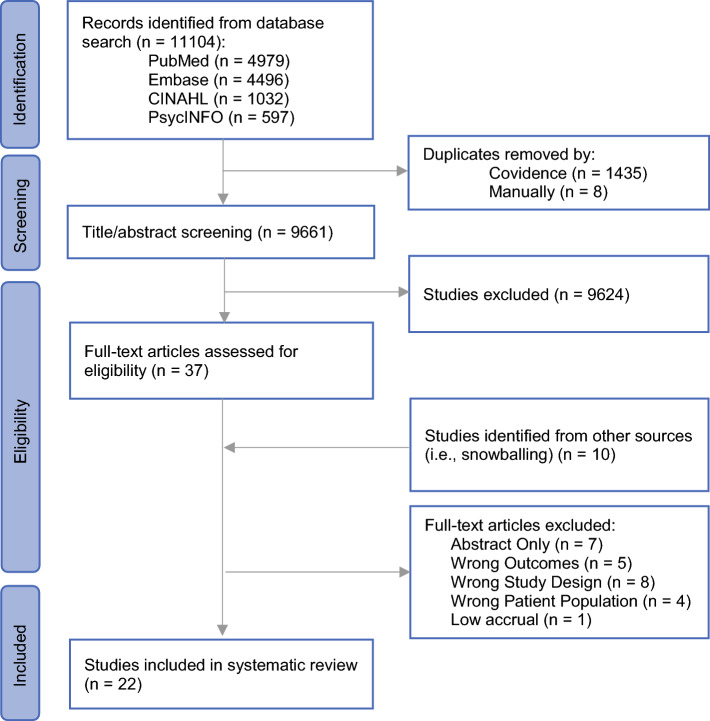
PRISMA figure

Among the 11,104 publications identified in the initial query, 1443 were identified as duplicates, and 9661 underwent title and abstract screening. After a full-text review of 37 articles, 15 studies were deemed eligible for inclusion. In addition, 10 studies were identified through the snowball procedure, 7 of which met the inclusion criteria. Overall, results were extracted from 22 studies that met the inclusion criteria (Fig. 1). The literature search identified five randomized control trials,^13–17^ eight cross-sectional studies,^18–25^ three cohort studies,^26–28^ three qualitative studies,^29–31^ two mixed-method studies,^32,33^ and one case-control study^34^ that evaluated surveillance-related anxiety after curative-intent surgery (Table 1).

**Table 1:** Summary of included studies

Study	Year	Country	No. of participants	Age (years)	Percentage female	Cancer type	Cancer stage	Procedure	Time since surgery at time of recruitment	Adjuvant therapy during assessment	Surveillance type
*RCT*											
Brown et al.	2002	UK	61 (C: 31, I: 30)	C: 63 (48–83)^a^ I: 68 (53–87)	100	Breast	AJCC I	Lumpectomy or mastectomy with or without axillary excision	C: average of 47 months I: average of 50 months	C: tamoxifen (64.5%) I: tamoxifen (63.3%)	Mammogram
Zhan et al.	2017	Netherlands	1591	68 (26–94)^b^	43.1	Colorectal	AJCC I–III	Curative-intent resection	After surgery or completion of adjuvant therapy	Completed before assessment	Clinical exam, CEA blood test, CT scan
Jeppesen et al.	2018	Denmark	212 (C: 77, I: 79)	C: 66.5 ± 8.9^c^ I: 63.4 ± 8.3	100	Endometrial	FIGO I	Hysterectomy	After surgery and pathologic stage confirmation	Participants receiving adjuvant therapy were excluded	Clinical exam, pelvic ultrasound
Naeser et al.	2022	Sweden	204 (C: 105, I: 99)	C: 67 (56–73)^d^ I: 61 (50–71)	C: 41 I: 37	Melanoma	AJCC IIB–III	Radical surgery	After surgery and pathologic stage confirmation	C: systemic (22%) I: systemic (19%)	Clinical exam, ultrasound, whole-body imaging, S-100B blood test
Ackermann et al.	2022	Australia	100 (C: 51, I: 49)	C: 59.7 ± 11.6^c^ I: 57.5 ± 12.3	C: 47 I: 45	Melanoma	AJCC 0–II	Curative-intent resection	After surgery and pathologic stage confirmation	NR	Clinical exam with dermatoscope
*Case-control*											
Porter et al.	2003	USA	55 (C: 21, Ca: 34)	C: 54.4 ± 10.0^c^ Ca: 56.3 ± 10.8	100	Breast	AJCC 0–III	Lumpectomy or Mastectomy	Cases: average 3.9 years	Cases: tamoxifen (68.8%)	Mammogram
*Cohort*											
Shelby et al.	2012	USA	204	59.5 ± 10.9^c^	100	Breast	AJCC I–IIIA	Lumpectomy or mastectomy	Average 5.01 years	NR	Mammogram
McGinty et al.	2016	USA	160	61.48 ± 9.6^c^	100	Breast	AJCC 0–IIIA	Lumpectomy or mastectomy	Completed treatment within past 3 years	Hormone therapy 79.87%	Mammogram
Elliott et al.	2023	Ireland, Sweden, UK	4682	64.3 ± 9.8^c^	22.4	Esophageal	AJCC 0–IVA	Extended total gastrectomy or esophagectomy	After surgery and pathologic stage confirmation	Not specified	Clinical exam, CT scan
*Cross-sectional*											
Kiebert et al.	1993	Netherlands	127 (G1: 67, G2: 60)	G1: 57 ± 13^c^ G2: 54 ± 12	G1: 67 G2: 78	Multiple	NR	Curative-intent resection	G1: <1 year (34%), 1–5 years (46%) G2: <1 year (32%), 1–5 years (48%)	NR	Clinical exam
Stiggelbout et al.	1997	Netherlands	130	68 ± 11^c^	49	Colorectal	NR	Curative-intent resection	Completed treatment within past 5 years	NR	Clinical exam
Papagrigoriadis et al.	2003	UK	95	NR	NR	Colorectal	NR	Curative-intent resection	<3 Years ago (63%), >5 years (4%)	Current therapy 3%	Clinical exam, colonoscopy
Kew et al.	2009	UK	96	58 (29–88)^b^	100	Gynecologic	NR	Curative-intent resection (94%)	Median 42 months	NR	Clinical exam
Greimel et al.	2011	Austria	210	61 ± 13^c^	100	Gynecologic	FIGO I–IV	Curative-intent resection (93%)	Median 44 months	NR	Clinical exam, blood test, pelvic ultrasound, CT, MRI, PET
Kelly et al.	2019	US	212	58.1 ± 13.5^c^	57.1	Multiple	NR	Curative-intent resection	Average 4.95 years	Chemotherapy (6.6%), Radiation (1.9%) or other (6.6%)	Clinical exam, imaging
Strausser et al.	2022	USA	337	G1: 61 (54–69)^d^ G2: 65 (46–85) G3: 69 (41–89)	21.1	Urologic	AJCC Tis-T3	Partial or radial nephrectomy, prostatectomy, TURBT, partial or full cystectomy	At least 60 days after surgery	NR	Clinical exam, imaging
Tepper et al.	2022	USA	65	<65: 69.2% ≥65:30.8%	52.3	Sarcoma	NR	Curative-intent resection	Median 385 days	NR	CT scan, x-ray
*Mixed methods*											
Koo et al.	2017	USA	12	71.8 (66–77)^e^	17	Bladder	AJCC Ta–Tis	Curative-intent resection	Average 6.5 years post-diagnosis	NR	Cystoscopy
Soriano et al.	2019	USA	57	58 ± 9^c^	100	Breast	AJCC 0–IIIA	Curative-intent resection	Average 12.2 months post-diagnosis	NR	Mammogram
*Qualitative*											
Sterba et al.	2015	USA	22	63 (43–86)^a^	55	Colorectal	AJCC I–III	Curative-intent resection	At least 2–18 months after treatment	NR	Clinical exam, blood tests, colonoscopy
Brandzel et al.	2017	USA	41	38–75	100	Breast	AJCC 0-III	Curative-intent resection	Completed treatment with exception of hormonal therapy	NR	Clinical exam, mammogram,, MRI
Harrison et al.	2023	UK	14	40–79	57.1	Kidney	NR	Curative-intent resection	78.6% within 2 years from surgery	NR	Clinical exam, CT scan

RCT, Randomized controlled trial; UK, United Kingdom; C, control group; I, intervention group; AJCC, American Joint Committee on Cancer; CEA, carcinoembryonic antigen; CT, computed tomography; FIGO, International Federation of Gynecology and Obstetrics; NR, not recorded; USA, United States; Ca, case group; G1, group 1; G2, group 2; MRI, magnetic resonance imaging; PET, positron emission tomography

^a^Mean with range

^b^Median with range

^c^Mean with standard deviation

^d^Median with interquartile range

^e^Mean with interquartile range

The majority of the studies were performed in the United States (*n* = 10), the United Kingdom (*n* = 5), and the Netherlands (*n* = 3). The most frequently included cancers were breast (*n* = 6) and colorectal (*n* = 4) cancers. Most of the studies did not specify the specific type of surgery performed before surveillance (*n* = 14). Overall, 8693 patients were included, the majority of whom were women, with nine studies focused solely on female-specific cancers. The mean age of participants, reported by 14 studies, was 61.9 years (range, 20–94 years).

The studies reported the use of many different types of surveillance, including physical examinations (e.g., dermatoscopic, full-body, gynecologic), laboratory tests (e.g., carcinoembryonic antigen [CEA]), and imaging tests (e.g., computed tomography [CT], magnetic resonance imaging [MRI]). Nine studies included more invasive surveillance evaluations, including mammograms, pelvic ultrasound, colonoscopy, and cystoscopy.

### Quality Assessment

With regard to the quality appraisal, 18 studies (82%) were of high quality, whereas four studies (18%) were assessed to be of moderate quality (Table S1). Among the randomized trials, the most common limitation to quality was the lack of double-blinding. In addition, it was unclear in two studies whether the assessors of outcomes were blind to treatment assignment. Many of the cross-sectional studies lacked valid and reliable study outcome measures and techniques to mitigate the impact of confounding variables. Finally, among the three qualitative and one mixed-methods study, the most common limitation to quality was attributed to not identifying the influence of researchers on the research.

### Qualitative and Mixed-Methods Studies

Overall, five studies used qualitative methods, including two studies with a mixed-methods approach.^32,33^ Data were gathered through focus group interviews (*n* = 3),^29–32^ semi-structured one-on-one interviews (*n* = 1),^30^ or daily diaries.^33^ Only the mixed-methods studies specified the timing of assessments to surveillance, with Koo et al. focusing on the time immediately after cystoscopy and Soriano et al.^32^ using daily diary entries beginning 2 weeks before the surveillance mammogram.^32^ Whereas some of the studies^29,31,32^ asked participants to recall their experiences specifically with surveillance-related anxiety, others probed with questions only about general attitudes and experiences surrounding follow-up care.^30^

Three of the qualitative studies reported anxiety and apprehension surrounding follow-up appointments as a universal theme in their qualitative analysis.^29,31,32^ Across all of the qualitative studies, the waiting period between the scan procedure and receipt of results was noted as a source of anxiety and psychological distress. Whereas some patients attributed their scanxiety to uncomfortable scan procedures (e.g., claustrophobia, cold surfaces),^29^ others cited the constant FCR.^31,32^ In addition, for some patients, the scan experience triggered negative emotions that they had experienced during the initial cancer diagnosis and treatment.^29,30^

However, not all feelings related to surveillance were negative, as demonstrated in the study by Koo et al.^32^ The participants in this study disclosed that scans reduced anxiety regarding recurrence. Some patients considered scans as a general confirmation that their treatment was effective and that they remained cancer-free.

### Quantitative Interventional Studies

Five randomized controlled trials (RCTs) evaluated the effect of an intervention on scanxiety. Four were randomized with a control group,^13,15–17^ whereas one used a stepped-wedge design.^14^ In all the trials, the control group included standard usual surveillance care, whereas the intervention involved some modification to the frequency or type of surveillance. In three trials, the participants were educated on alarming symptoms and provided options for self-referral.^13,15,17^

The instruments used to measure scanxiety varied, but the Hospital Anxiety and Depression Scale was the most common (Table 2). The overall rates of measured scanxiety were low, although one study on endometrial cancer noted that 20% of all the study participants experienced clinically significant FCR around the time of follow-up evaluation.^15^ Four of the studies reported no significant differences in scanxiety between the control groups and modified surveillance groups, with Jeppesen et al.^15^ finding that traditional follow-up methods reduced anxiety more effectively than patient-initiated follow-up methods (Table 2).

**Table 2:** Outcomes of included randomized controlled trials

Study	Allocation	Control	Intervention	Assessment time point	Assessment tools	Conclusions
Brown et al.	Parallel-group randomization	Standard follow-up care: visit frequency not specified	Patient-initiated follow-up with yearly mammogram and written information on signs and symptoms of recurrence	Baseline, 6 months, and 1 year	(1) EORTC QLQ-C30 and QLQ-BR23 (2) HAD scale (3) Structured interviews	(1) No significant differences in quality of life or psychological morbidity (2) Standard clinic follow-up provided reassurance, whereas patient-initiated follow-up provided convenience
Zhan et al.	Stepped wedge cluster randomization	Standard follow-up care: every 3–6 months CEA measurement and clinic visit every 6 months in years 1–3, annual CEA and visit in years 4–5	Bimonthly CEA measurements in years 1–3, trimonthly in years 4–5 combined with CT imaging	First time point: 3/5 clusters transitioned to intensified protocol Second time point: all clusters in intensified protocol	(1) Validated 16-item questionnaire on attitude toward follow-up (2) Validated 3-item questionnaire on fear of recurrence, cancer worry scale, HAD scale (3) 15-item self-developed questionnaire on experiences and expectations	(1) No significant differences in attitude toward follow-up, anxiety and depression, fear of recurrences, and cancer worries between the intensified follow-up protocol and care as usual (2) Patients had improved responses to intensified protocol during the second measurement, suggesting adaptation to new protocol
Jeppesen et al.	Parallel-group randomization	Standard follow-up care: scheduled visits every 4–6 months in first 2 years, every 6 months in year 3	Patient initiated follow-up with education of alarm symptoms	1, 3, 6, and 10 months of follow-up	(1) Fear of cancer recurrence inventory	(1) Traditional follow-up alleviated fear of cancer recurrence more effectively than patient-initiated follow-up (2) Intervention group had significantly fewer hospital examinations
Naeser et al.	Parallel-group randomization	Standard follow-up care: examinations every 12 months for stage II and every 6 months for stage III, ultrasound every 6 months for positive sentinel node(s), whole-body imaging every 6 months if receiving adjuvant therapy	Standard follow-up plus whole-body imaging and S-100B blood test at baseline, 6, 12, 24, and 36 months	Baseline and 1 year	(1) EORTC QLQ-C30 (2) HAD scale	(1) No significant differences in quality of life or anxiety and depressive symptoms between the two study arms at baseline or 1-year follow-up (2) Levels of anxiety and depressive symptoms were generally low in the study group
Ackermann et al.	Parallel-group randomization	Standard follow-up care: scheduled visits as per treating clinician	Standard follow-up plus provided personal dermatoscope and web-based skin checker application for self-skin exams every 2 months	Baseline and 6 months	(1) Melanoma-specific fear of cancer recurrence inventory (2) DASS-21	(1) No evidence of a difference in psychological outcomes between study arms (2) Patient-led surveillance was safe and feasible

EORTC, European Organization for Research and Treatment of Cancer; QLQ-C30; QLQ-BR23; HAD, Hospital Anxiety and Depression Scale; CEA, carcinoembryonic antigen; DASS, Depression Anxiety and Stress Scales

Two trials also assessed participants’ attitudes toward the interventions. In the study assessing the self-management approach to surveillance after treatment for endometrial cancer, more women in the control group reported feeling reassured.^13^ These women endorsed a sense of relief when a health care professional conducted the exam and confirmed no recurrence. In contrast, more intervention group participants felt having a self-management aspect was convenient. In the study assessing intensified surveillance (i.e., frequent CEA measurements), colorectal cancer survivors found frequent blood tests more stressful, less reassuring, and more inconvenient.^14^ However, this anxiety subsided over time, suggesting adaptation to the intervention.

### Quantitative Non-Interventional Studies

Overall, this review included 12 more non-interventional studies in addition to the 2 aforementioned mixed-methods studies. These studies used a wide range of assessment tools, with seven utilizing general measures^18,19,21,23,27,28,33^ that assessed psychological symptoms without making any explicit reference to imaging, and the other half using scan-specific measures.^20,22,24–26,32,34^ As such, these tools measured varying psychological symptoms including anxiety, depression, distress, FCR, and emotional well-being, among others. The studies used 29 unique measures with study-adapted Likert scales, patient-reported outcomes Measurement Information System Short-Form Emotional Distress-Anxiety. The most common measure was the Psychological Consequences Questionnaire (Table 3).

**Table 3:** Outcomes of included case-control, cohort, cross-sectional, mixed methods, and qualitative studies

Study	Comparison groups	Assessment time point	Assessment tools	Conclusions
*Case-control*				
Porter et al.	Case: breast cancer survivors Control: women with no history of cancer scheduled for routine mammogram	(1) Phase 1: 1 month before mammogram, (2) Phase 2: day before, day of, and day after mammogram	(1) Validated assessment of mood (2) Daily stress scale (3) Psychological consequences Questionnaire Salivary cortisol	(1) No significant differences noted in stress and mood between groups at both baseline and phase 2 (2) Among cancer survivors, there were no significant associations between cortisol measures and general stress ratings.
*Cohort*				
Shelby et al.		Immediately before and after mammogram	(1) Modified Stanford Acute Stress Reaction Questionnaire (2) Brief Pain Inventory, Pain Catastrophizing Scale	(1) Higher levels of mammography-related anxiety and pain catastrophizing were associated with not returning for a mammogram. (2) Mammography-pain was not associated with adherence.
McGinty et al.		(1) One month, 1 week and immediately before mammogram (2) Immediately after, 1 week, and 1 month after receiving results	(1) Two visual analogue scales assessing fear of recurrence Cancer Worry Scale	(1) Fear of recurrence varied significantly over time: scores increased before the scan, decreased as soon as the results of the mammography were negative, and increased again within one-month post-scan. (2) Breast cancer patients who reported greater perceived risk, lower coping self-efficacy, and greater reassurance-seeking behavior were associated with higher fear of recurrence.
Elliott et al.	Control: esophageal cancer survivors undergoing standard follow-up Intervention: intensive surveillance (annual CT for 3 years post-operatively)	At least 1 year after completion of treatment	EORTC QLQ-C30 and QLQ-OG25	(1) Patients undergoing intensive surveillance exhibited greater anxiety scores in comparison with standard follow-up. (2) Intensive surveillance was not associated with global health status, emotional functioning, or financial difficulties.
*Cross-sectional*				
Kiebert et al.	Two groups based on time of survey assessment	(1) Group 1: 1 month before follow-up (2) Group 2: At follow-up visit and 2 weeks later	(1) Self-developed 13-item questionnaire on attitudes toward follow-up and fear of recurrence (2) Rotterdam Symptom Check List, quality-of-life scale	(1) In the month before follow-up, patients viewed follow-up as burdensome and reported more sleep disturbances. (2) There was no significant difference in physical or psychological distress between patients at time of follow-up and 1 month before. (3) There was no difference in the overall quality of life 1 month before, on the day of the follow up visit, and 2 weeks later.
Stiggelbout et al.	Three groups based on time of survey assessment	(1) Group 1: 1 week before follow-up (2) Group 2: 2 weeks after a follow-up (3) Group 3: between two follow-up visits	(1) Medical Outcomes Study Short-Form General Health Survey (2) Rotterdam Symptom Checklist (3) Visual analogue scale assessing quality of life (4) Validated 16-Item Questionnaire on attitudes toward follow-up	(1) No association between follow-up visits and quality of life (2) Patients had greater positive attitudes toward follow-up, with communication with physician and reassurance significantly outscoring nervous anticipation and general disadvantages.
Papagrigoriadis et al.		No relation to surveillance encounter	Self-designed 39-item questionnaire on attitudes toward follow-up and investigations	(1) Appointment proximity caused anxiety, sleep problems, and decreased appetite. (2) Majority of patients felt reassured and optimistic for the future after receiving results.
Kew et al.		At time of follow-up appointment	Self-designed 11-item questionnaire on attitudes toward follow-up	(1) More than half of women reported feeling more anxious before attending a follow-up visit with more than one third reporting relief after a visit. (2) Detection of recurrence was ranked by participants as the most important reason for follow-up.
Greimel et al.		At time of follow-up appointment	(1) Breast Cancer – Psychological Assessment Screening Scale (2) Self-designed questionnaire on attitudes and needs for follow-up	(1) Majority of patients showed low levels of emotional distress, with two thirds of patients reporting low levels of anxiety before follow-up. (2) Less than one third of patients reported moderate worries before attending the hospital for routine follow-up.
Kelly et al.	Four groups stratified based on overall self-reported well-being including financial toxicity	At time of follow-up appointment	(1) Comprehensive Score for Financial Toxicity (2) General Functional Assessment of Cancer Therapy Questionnaire (3) Attitudes Towards Follow-Up Scale	(1) Participants with the highest level of well-being felt more reassured by follow-up visits. (2) Conversely, patients with the lowest sense of well-being had the highest level of nervousness related to follow-up visits.
Strausser et al.		Before revealing test results	Adapted 23-item questionnaire on attitudes toward follow-up	(1) Majority of patients reported that follow-up visits provided a sense of security and did not consider diagnostic tests to be burdensome. (2) One fifth of patients reported wanting strategies to combat fear and anxiety around follow-up visits.
Tepper et al.		At time of follow-up appointment	(1) Self-developed sarcoma surveillance survey (2) Appraisal scale (3) Patient-reported outcomes Measurement Information System Short-Form Emotional Distress-Anxiety (PROMIS)	(1) Approximately one third of patients identified cost and radiation exposure as concerns regarding surveillance imaging. (2) Higher levels of anxiety were correlated with preference for more frequent surveillance and overall level of concern regarding follow-up visits.
*Mixed methods*				
Koo et al.		After completion of cystoscopy	(1) Psychological Consequences of Screening Questionnaire (PCQ) (2) Customer Satisfaction Survey (CSS) (3) Focus Groups	(1) Approximately two thirds of patients reported some degree of pre-procedural anxiety and worry. (2) Both survey and qualitative results demonstrated that participants experienced improved well-being after surveillance.
Soriano et al.		(1) Survey 3 weeks before mammogram (2) 3-week daily diary starting 2 weeks before mammogram	Behavioral Inhibition System (BIS) Scale (2) PROMIS Anxiety (3) Modified Fear of Cancer Recurrence Inventory	(1) Fear of recurrence increased, leading to mammogram, but was not well-predicted with participants’ global anxiety or personality factors. (2) Caretaker personality characteristics may influence fear of recurrence in patients.
*Qualitative*				
Sterba et al.		No relation to surveillance encounter	Focus group or individual semi-structured interview	(1) Fear of surveillance tests and long intervals between follow-up visits were identified as negative experiences associated with surveillance (2) Trust in healthcare providers, symptom management, and reassurance were identified as motivators for follow-up
Brandzel et al.		No relation to surveillance encounter	Focus Groups	(1) Anxiety associated with surveillance scans was attributed to fear of recurrence and remembrance of initial breast cancer diagnosis (2) Additional negative aspects of surveillance imaging were the discomfort of undergoing a mammogram and its associated financial costs
Harrison et al.		No relation to surveillance encounter	Focus Groups	(1) Anxiety about follow-up appointments was a universal experience of the focus group attendees (2) Specific concerns that increased stress included difficulty with scheduling appointments and a prolonged duration between scans and receiving results

### Prevalence and Severity

Given the heterogeneity of the assessment tools, the overall prevalence and severity of scanxiety could not be summarized. However, in the four studies reporting prevalence, 34–85% of the participants experienced some anxiety surrounding follow-up visits.^21,22,26,32^ For example, by assessing anticipatory anxiety immediately before and after a mammogram, Shelby et al.^26^ observed that 85% of women experienced scanxiety, and one third reported three or more symptoms of anxiety. Conversely**,** in a study involving gynecologic cancer survivors, 20% reported moderate levels of scanxiety, and 14% had severe levels. In addition, about one third of the survivors also reported moderate worries.^22^ In numerous studies, the patients reported experiencing increased somatic symptoms such as sleep disturbances, irritability, worry, dizziness, poor concentration, fatigue, depressed feelings, and lack of energy.^18,20,24,26^ In a study of esophageal cancer survivors, those undergoing intensive surveillance had greater anxiety on the QLQ-OG25 than those undergoing standard follow-up procedures.^28^

### Timing of Symptoms

Six of the non-interventional studies that used quantitative measures assessed the impact of time by comparing scanxiety at varying points in the scan experience. Four studies^21–23,25^ assessed scanxiety at the time of follow-up appointment, whereas the remaining studies assessed scanxiety in the scan-to-results period.^24,32^ In four studies with a longitudinal design, stress^18,34^ and FCR^27,33^ increased leading up to the scan and decreased after patients received results on the day of their follow-up visit. Moreover, in two studies with single assessment time points, more than half of the participants reported feeling more anxious before attending a follow-up visit, with more than one third reporting relief^21^ and improved well-being^32^ after surveillance. Consistent with these findings, colorectal cancer survivors reported that appointment proximity caused anxiety, sleep problems, and decreased appetite.^20^ The results from these studies also indicated that the participants experienced more sleep disturbances in the month before the follow-up visit,^18^ as well as some irritability and intrusive thoughts several days before the follow-up visit.^22^

### Factors Associated With Scanxiety

Several of the included studies reported factors associated with scanxiety such as sociodemographic and cancer-related characteristics, overall well-being, scan adherence, coping self-efficacy, and other concerns. One study found that women, breast cancer survivors, and patients with a lower standard of education experienced more psychological distress than men, patients with other malignancies, and patients with higher education.^18^ In a study analyzing the association between patient-reported well-being (i.e., social/family, emotional, functional) and scanxiety, the patients with the lowest level of well-being exhibited the highest level of nervousness and anxiety before the follow-up appointment. In contrast, the patients with the highest level of well-being felt more reassured by follow-up visits.^23^ In a study with breast cancer survivors, Shelby et al.^26^ reported that higher levels of mammography-related anxiety were associated with not returning for mammography in the following year compared with lower anxiety levels. In the same cohort, the women with higher levels of anticipatory anxiety also reported more negative responses to pain during the mammogram, potentially indicating a link between scan adherence and scanxiety.

An important factor associated with FCR was coping self-efficacy. For example, for one group of breast cancer survivors, the authors concluded an inverse relationship between FCR and coping self-efficacy. More specifically, women with a lower confidence in their ability to cope with a potential recurrence experience had a higher FCR around their surveillance mammography.^27^ Concerns about associated cost and radiation exposure from the surveillance imaging were noted by nearly one third of the patients in one study.^25^ In addition, the patients with higher levels of anxiety reported a greater preference for more frequent surveillance imaging. None of the three studies examining quality of life demonstrated an association between overall quality of life and scanxiety.^18,19,28^

### Positive Aspects of Surveillance

Several studies reported participants expressing a positive attitude because tests provided relief and reassurance. For example, Stiggelbout et al.^19^ observed that a large majority of patients had greater positive attitudes toward follow-up visits, with communication with physicians and reassurance significantly outscoring nervous anticipation and general disadvantages. Similarly, in another study with an assessment tool used before test results were revealed, the majority of the patients reported that follow-up visits provided a sense of security, whereas one fifth wanted strategies to combat fear and anxiety around surveillance visits.^24^ In addition, some expressed feeling optimistic after receipt of the results.^20^

## Discussion

Postoperative surveillance is a critical part of cancer survivorship care. However, given the possibility that surveillance may identify a cancer recurrence, follow-up visits may be a significant source of anxiety and distress for many cancer survivors. Previous systematic reviews have characterized scan-associated anxiety during screening, cancer treatment assessment, and active surveillance, but less research has focused on scanxiety experienced by patients after curative-intent surgery. The results from this systematic review highlight that although scanxiety is nearly universal across multiple cancer types and patient populations, it is transient and generally limited in severity. At the same time, existing literature on interventions to reduce scanxiety has not identified effective strategies to date.

The reported prevalence of scanxiety was non-uniform across studies, with qualitative studies showing universal scanxiety and quantitative studies reporting mixed findings. This may be explained in part by the differences in assessment tools and heterogeneity of the study population. In addition, heterogeneity of surveillance types also may be a factor because each scan method introduces varying degrees of discomfort. For example, in one study, lung cancer patients reported greater anxiety, discomfort, and claustrophobia when undergoing MRI scans rather than CT scans.^35^

Several studies attempted to identify potential predictors of who may be more susceptible to scanxiety, including, but not limited to, cortisol measurements, mental health status, overall well-being, financial well-being, and caretaker characteristics. Consistent with the findings of Kiebert et al.,^18^ previous literature on advanced cancer patients shows that those with lower education levels may be more likely to experience scanxiety.^36^ Kiebert et al.^18^ also reported greater severity of scanxiety among women, which is consistent with existing literature on advanced populations.^7,37^ In addition, one study in this review reported that women with higher levels of mammography-related anxiety also reported higher pain catastrophizing.^26^ Moreover, in a prior scoping review, numerous studies focused on emotional experiences related to breast cancer screening reported a positive association between pain levels during mammography and scanxiety.^38^

For many, surveillance scans and follow-up visits felt like a reliving of the initial cancer diagnosis and treatment, whereas for others, scan procedures felt claustrophobic, were uncomfortable, and elicited concerns regarding radiation exposure and financial costs. In both qualitative and quantitative studies, participants reported the scan-to-results waiting period to be the most anxiety-provoking and that scanxiety in general dissipated after their results were shared. Although preferable to not be pervasive, the transient nature of scanxiety for most patients could represent challenges to the development of effective interventions to mitigate its impact in the future. Notably, numerous studies have reported that patients had positive attitudes toward the follow-up visit because it provided opportunities for symptom management, sense of security, and reassurance.

A few studies evaluated the effect of modifying surveillance schedules, but no significant differences in scanxiety were identified. Overall, the impact of patient-initiated surveillance was mixed. As seen in a Brown et al.^13^ study, although this model leads to fewer clinic visits and enables patient empowerment without sacrificing missed cancer recurrence, it increases treatment burden on the patient and leads to increased anxiety. The responsibility for detecting signs of recurrence may lead to distress, and the complete absence of a scheduled hospital-based visit may forgo the opportunity to achieve medical reassurance, a key component in alleviating the FCR.^39^ Intensified surveillance also was shown to have no significant impact on anxiety compared with usual care. This suggests that the surveillance frequency may not be a significant factor influencing scanxiety.

Although we found no published studies in this systematic review, existing literature suggests that integrating education and emotional or psychological support may be a promising approach to reducing anxiety associated with medical testing. For example, in two studies of breast cancer screening, the patients were given psychological support in addition to screening information immediately before the scan.^40,41^ Both studies observed a reduction in scanxiety. These findings further support survivors’ desire for strategies to combat fear and anxiety around surveillance visits.^24^

Our findings highlight several potential areas for future research. First, there is a need to develop a universal scanxiety assessment tool to properly assess its prevalence and severity. This will provide further insight into differences in anxiety related to cancer and surveillance types.

Second, researchers should focus on identifying at-risk groups by potentially assessing patients at the time of diagnostic screening or using other potential identifiers not considered in the included studies (e.g., being treated for anxiety/depression, lack of support system). Although previous studies have identified effective strategies to mitigate scanxiety in screening populations, none have found interventions for patients during the postoperative surveillance period.

Third, given the rapidly rising population of cancer survivors, effective strategies to reduce scanxiety and improve quality of life are needed. Interventions aimed at modifying the surveillance type and frequency, as outlined in the current review, have not been effective at reducing scanxiety. The data in our review suggest that the optimal timing of intervention is before the scan-to-results waiting period.^3,36,37^ Incorporation of supportive care interventions with routine follow-up visits may be beneficial because it allows opportunities for pain management, addressing psychological symptoms, clarifying patient preferences, and facilitating shared decision-making.^42^ Given that formal supportive care services (e.g., palliative care, psychosocial oncology) are constrained at many institutions, training oncology providers to support the care needs of patients directly with low levels of scanxiety (i.e., primary supportive care) could be an alternative that supports the survivorship needs of most patients.

Future work also should focus on understanding risk factors for scanxiety so that supportive care resources can be targeted toward those patients at highest risk for severe surveillance-associated distress. This review also supports the potential effectiveness of teaching self-management strategies such as self-regulatory skills to optimize coping self-efficacy and outcome expectations to manage FCR.

This systematic review had several limitations, largely due to the quality of evidence and the heterogeneity of the studies included. For example, scanxiety was defined broadly in the current review, with consequent heterogeneity in its definition and measurement. Study design varied, and the diversity of assessment points in longitudinal studies was large, limiting the ability to summarize the development of scanxiety and closely related concepts (e.g., FCR) throughout the survivorship period. This limitation prevented us from statistically pooling the results of the included studies, thereby preventing us from carrying out a meta-analysis. Moreover, details regarding assessment time points as well as clinical variables (e.g., cancer stage, surveillance method) and demographic variables (e.g., race, ethnicity, income) were missing from numerous studies. As such, we could not draw firm conclusions on the potential factors associated with greater scanxiety. Finally, no studies evaluated the impact of direct patient access to test results via the electronic health record on scanxiety, which will be an important area of future research.^43,44^

Despite these limitations, the current systematic review of surveillance-associated anxiety after curative-intent surgery found that scanxiety is near universally experienced but generally limited in severity and duration. Additional research is needed to confirm which patient populations are at highest risk for scanxiety and to guide the development and delivery of interventions aimed at mitigating distress and improving quality of life among cancer survivors.

## Supplementary Information

Below is the link to the electronic supplementary material.

Supplementary file1 (DOCX 42 KB)
