# Supplementary material for: Surveillance-Associated Anxiety After Curative-Intent Cancer Surgery: A Systematic Review
Source: Ann Surg Oncol. 2024 Sep 29;32(1):47–62. doi: 10.1245/s10434-024-16287-5 (PMC11659363; doi:10.1245/s10434-024-16287-5)
Supplement: Supplementary file 1 — Supplementary file1 (DOCX 42 KB) [file 10434_2024_16287_MOESM1_ESM.docx]

**Surveillance-Associated Anxiety Following Curative-Intent Cancer Surgery: A Systematic Review**

Rakhsha Khatri, BA;^1^ Patrick L. Quinn, MD;^1^ Sharla Wells-Di Gregorio, PhD;^2^ Timothy M. Pawlik, MD, PhD, MPH; ^1^ Jordan M Cloyd, MD^1^

1. The Ohio State University Wexner Medical Center, Division of Surgical Oncology, Department of Surgery, Columbus, Ohio, USA
2. The Ohio State University Wexner Medical Center, Division of Palliative Medicine, Department of Internal Medicine, Columbus, Ohio, USA

**Shortened Title**: Scanxiety after Cancer Surgery

**Disclosure Statement:** The authors have no conflicts of interest to report.

**Corresponding Author:**

Jordan M. Cloyd

Associate Professor

Department of Surgery

The Ohio State University Wexner Medical Center

410 W 10^th^ Ave

Columbus OH 43210

Jordan.Cloyd@osumc.edu

Phone: 614-730-4225

Fax: 614-293-3465

**Supplementary Table 1:** Representative search string used in PubMed, Embase, CINAHL and PsycINFO searches for studies pertaining to scan-associated anxiety after curative intent surgery.

**PubMed | Results:** 4,979

| 1 | (Anxiet*[tiab] OR "anxiety"[Mesh] OR “scanxiety” OR “anxiety disorders”[Mesh] OR “Scan-associated anxiety”[tiab] OR “scan-associated distress”[tiab] OR agitat*[tiab] OR "anticipatory anxiety"[tiab] OR “Hypervigilant”[tiab] OR “hypervigilance”[tiab] OR “Nervous”[tiab] OR “Nervousness”[tiab] OR “Anxiousness”[tiab] OR "Psychological Distress"[Mesh] OR Distress*[tiab] OR "Psychological Distresses"[tiab] OR "Distress, psychological" OR “Emotional Stresses”[tiab] OR “Emotional Distresses”[tiab] OR “Emotional Distress”[tiab] OR “Distress, Emotional”[tiab] OR Stress*[tiab] OR “Emotional Stress”[tiab] OR “Stress, Emotional”[tiab] OR “Fear”[MeSH] OR Fear*[tiab] OR Worr*[tiab] OR Dread*[tiab] OR “Apprehension”[tiab] OR “Psychological Stress”[tiab] OR "Stress, Psychological"[Mesh] OR "Stress, Psychological"[tiab] OR “Psychological Stresses”[tiab] OR “Stresses, Psychological”[tiab] OR “Stress, Psychologic”[tiab] OR “Psychologic Stress”[tiab] OR “Stressor, Psychological”[tiab] OR “Psychological Stressor”[tiab] OR “Psychological Stressors”[tiab] OR “Stressors, Psychological”[tiab] OR “Fear of recurrence”[tiab]) |
| --- | --- |
| 2 | ("surveillance"[tiab] OR "surveillances"[tiab] OR "surveilled"[tiab] OR "Monitor"[tiab] OR "monitoring"[tiab] OR "follow-up"[tiab] OR "follow ups"[tiab]) |
| 3 | (Cancer*[tiab] OR "neoplasms"[MeSH] OR neoplas*[tiab] OR "Carcinoma”[Mesh] OR carcinoma*[tiab] OR tumor*[tiab] OR tumour*[tiab] OR malignan*[tiab] OR “malignant neoplasm”[tiab] OR “malignant neoplasia”[tiab] OR sarcoma*[tiab] OR “Adenocarcinoma”[Mesh] OR Adenocarcinoma*[tiab]) |
| 4 | ("surgical procedures, operative"[Mesh] OR "surgical procedures, operative”[tiab] OR “operative surgical procedure”[tiab] OR “operative surgical procedures”[tiab] OR “Surgery”[tiab] OR “Surgeries”[tiab] OR “surgerys”[tiab] OR “Surgical”[tiab] OR “surgically”[tiab] OR “surgicals”[tiab] OR “operation”[tiab] OR “operations”[tiab] OR “operative”[tiab] OR resect*[tiab]) |
| 5 | 1 AND 2 AND 3 AND 4 AND 5 |
| 6 | Limit 5 to humans and English |

**Embase** | Results: 4,496

| 1 | ('anxiety'/exp OR 'anxiety' OR 'anxieties' OR 'anxiety disorder'/exp OR 'anxiety disorder' OR 'anticipatory anxiety' OR 'scanxiety' OR 'scan-associated anxiety' OR ‘hypervigilance’/exp OR 'nervousness'/exp OR 'nervous tension' OR anxious*.mp OR distress*.mp OR 'psychological distress' OR 'psychological distresses' OR 'emotional stress'/exp OR 'emotional exhaustion' OR 'emotional stress' OR 'emotional tension' OR stress*.mp OR 'emotional distress' OR 'distress, emotional' OR 'emotional distresses' OR 'fear'/exp OR fear*.mp OR 'patient worry'/exp OR 'patient worry' OR worr*.mp OR dread*.mp OR 'apprehension' OR 'mental stress'/exp OR 'mental tension' OR 'nervous stress' OR 'psychic tension' OR 'psychosocial stress' OR 'tension, mental' OR 'tension, psychic' OR 'fear of recurrence') |
| --- | --- |
| 2 | ('disease surveillance'/exp OR 'monitoring'/exp OR monitor*.mp OR surveillance.mp OR 'follow up'/exp OR 'follow up' OR followup.mp OR 'follow ups') |
| 3 | (‘neoplasm’/exp OR neoplas*.mp OR cancer*.mp OR tumor*.mp OR tumour*.mp OR malignan*.mp OR ‘malignant neoplasm’/exp OR oncolog*.mp OR carcinoma*.mp OR 'carcinoma'/exp OR 'adenocarcinoma'/exp OR adenocarcinoma*.mp OR ‘sarcoma’/exp OR ‘malignant neoplas*’) |
| 4 | ('surgery'/exp OR surger*.mp OR 'surgical treatment' OR ‘perioperative period’/exp OR perioperative.mp OR intraoperative.mp OR 'surgical removal' OR 'operative surgical procedure*' OR surgical*.mp OR operat*.mp OR resect*.mp OR ‘resection’) |
| 5 | 1 AND 2 AND 3 AND 4 |
| 6 | Limit 5 to humans and English |

**CINAHL** | Results: 1,032

| 1 | ((MH "Anticipatory Anxiety") OR (MH "Anxiety/PC") OR (MH "Fear/PC") OR (MH "Worry") OR (MH "Psychological Distress") OR (MH "Stress, Psychological") OR (MH "Emotions") OR Anxiet* OR Scanxiety OR “Scan-associated anxiety” OR Hypervigilance OR Nervous OR Nervousness OR Anxious OR Anxiousness OR "Psychological Distress" OR Agitat* OR distress* OR “Psychological Distresses” OR “Distress, psychological” or “Distresses, psychological” OR “Emotional Distress” OR “Emotional Distresses” OR “Distress, Emotional” OR “Distresses, Emotional” OR Stress OR “Emotional Stress” OR “Emotional Stresses” OR “Stress, Emotional” OR Fear* OR Worr* OR dread* OR Apprehension OR “Psychological Stress” OR "Stress, Psychological" OR “Psychological Stresses” OR “Stresses, Psychological” OR “Stress, Psychologic” OR “Psychologic Stress” OR “Stressor, Psychological” OR “Psychological Stressor” OR “Psychological Stressors” OR “Stressors, Psychological” OR “Fear of recurrence”) |
| --- | --- |
| 2 | ((MH "Disease Surveillance") OR "surveillance" OR "surveillances" OR "surveilled" OR “Monitor” OR “Monitoring” OR “follow-up” OR “follow ups” OR “Recurrence”) |
| 3 | ((MH "Neoplasms") OR (MH "Carcinoma") OR (MH "Adenocarcinoma”) OR (MH “Sarcoma”) OR Cancer* OR neoplasm* OR neoplasia* OR tumor* OR tumour* OR malignant OR malignancy OR malignancies OR carcinoma OR “malignant neoplasm” OR “malignant neoplasms” OR “malignant neoplasia” OR sarcoma OR Adenocarcinoma) |
| 4 | ((MH "Surgery, Operative") OR (MH "Invasive Procedures") OR "surgical procedures, operative" OR “operative surgical procedure” OR “operative surgical procedures” OR “Surgery” OR “Surgeries” OR “surgerys” OR “Surgical” OR “surgically” OR “surgicals” OR operation OR operative OR “operate” OR “operating” OR resect* OR “resection”) |
| 5 | 1 AND 2 AND 3 AND 4 |
| 6 | Limit 5 to humans and English |

**PsycINFO** | Results: 597

| 1 | (DE "Anxiety") OR (DE "Anxiety Disorders") OR (DE "Fear") OR (DE "Stress") OR (DE "Psychological Stress") OR Anxiety OR Anxieties OR “Anticipatory anxiety” OR Scanxiety OR Hypervigilance OR Angst OR Nervous OR Nervousness OR Anxious OR Anxiousness OR Distress* OR "Psychological Distress" OR “Psychological Distresses” OR “Distress, psychological” OR “Emotional Distress” OR “Distress, Emotional” OR Stress* OR “Emotional Stress” OR “Emotional Stresses” OR “Stress, Emotional” OR Fear* OR Worr* OR Dread* OR Apprehension OR "Stress, Psychological" OR “Psychological Stresses” OR “Stresses, Psychological” OR “Stress, Psychologic” OR “Psychologic Stress” OR “Stressor, Psychological” OR “Psychological Stressor” OR “Psychological Stressors” OR “Stressors, Psychological” OR “Fear of recurrence”) |
| --- | --- |
| 2 | (DE "Disease Surveillance" OR DE "Monitoring" OR “surveillance” OR “surveillances” OR “surveilled” OR “Monitor” OR “Monitoring” OR “follow-up” OR “follow ups”) |
| 3 | (DE "Neoplasms" OR DE "Oncology" OR DE "Melanoma" OR Cancer* OR neoplasm* OR neoplasia* OR tumor* OR tumour* OR malignant OR malignancy OR malignancies OR carcinoma OR “malignant neoplasm” OR “malignant neoplasms” OR “malignant neoplasia” OR sarcoma OR Adenocarcinoma) |
| 4 | (DE "Surgery" OR "surgical procedures, operative" OR “operative surgical procedure” OR “operative surgical procedures” OR “Surgery” OR “Surgeries” OR “surgerys” OR “Surgical” OR “surgically” OR “surgicals” OR operation OR operative OR “operate” OR “operating” OR resect* OR “resection”) |
| 5 | 1 AND 2 AND 3 AND 4 |
| 6 | Limit 5 to humans and English |

**Quality Appraisal** | Y = Yes, N = No, U = Unclear, NA = Not applicable

**Supplementary Table 2**: Quality assessment using the Joanna Briggs Institute (JBI) Critical Appraisal Checklist for Randomized Control Trials.

| **Author** | **Q1** | **Q2** | **Q3** | **Q4** | **Q5** | **Q6** | **Q7** | **Q8** | **Q9** | **Q10** | **Q11** | **Q12** | **Q13** | **Total** | **Quality** |
| --- | --- | --- | --- | --- | --- | --- | --- | --- | --- | --- | --- | --- | --- | --- | --- |
| Ackermann | Y | Y | Y | N | N | Y | Y | N | Y | Y | Y | Y | Y | 77% | High |
| Jeppesen | Y | Y | Y | N | N | Y | Y | Y | Y | Y | Y | Y | Y | 85% | High |
| Naeser | Y | Y | Y | N | N | U | Y | Y | Y | Y | Y | Y | Y | 77% | High |
| Zhan | Y | Y | U | N | N | N | Y | Y | Y | Y | Y | Y | Y | 69% | Medium |
| Brown | U | U | Y | N | N | U | Y | Y | U | Y | Y | Y | Y | 54% | Medium |

*Q1: Was true randomization used for assignment of participants to treatment groups?*

*Q2: Was allocation to treatment groups concealed?*

*Q3: Were treatment groups similar at the baseline?*

*Q4: Were participants blind to treatment assignment?*

*Q5: Were those delivering treatment blind to treatment assignment?*

*Q6: Were outcomes assessors blind to treatment assignment?*

*Q7: Were treatment groups treated identically other than the intervention of interest?*

*Q8: Was follow up complete and if not, were differences between groups in terms of their follow up adequately described and analyzed?*

*Q9: Were participants analyzed in the groups to which they were randomized?*

*Q10: Were outcomes measured in the same way for treatment groups?*

*Q11: Were outcomes measured in a reliable way?*

*Q12: Was appropriate statistical analysis used?*

*Q13: Was the trial design appropriate, and any deviations from the standard RCT design (individual randomization, parallel groups) accounted for in the conduct and analysis of the trial?*

**Supplementary Table 3**: Quality assessment using the Joanna Briggs Institute (JBI) Critical Appraisal Checklist for cross-sectional studies.

| **Author** | **Q1** | **Q2** | **Q3** | **Q4** | **Q5** | **Q6** | **Q7** | **Q8** | **Total** | **Quality** |
| --- | --- | --- | --- | --- | --- | --- | --- | --- | --- | --- |
| Kelly | Y | Y | Y | Y | Y | Y | Y | Y | 100% | High |
| Stiggelbout | Y | Y | Y | Y | Y | N | N | Y | 75% | High |
| Kew | Y | Y | Y | Y | N | N | N | Y | 63% | Medium |
| Greimel | Y | Y | Y | Y | N | N | N | Y | 63% | Medium |
| Papagrigoriadis | Y | Y | Y | Y | Y | Y | N | Y | 88% | High |
| Strausser | Y | Y | Y | Y | Y | N | Y | Y | 88% | High |
| Tepper | Y | Y | Y | Y | Y | N | Y | Y | 88% | High |
| Kiebert | Y | Y | Y | Y | Y | Y | N | Y | 88% | High |

*Q1: Were the criteria for inclusion in the sample clearly defined?*

*Q2: Were the study subjects and the setting described in detail?*

*Q3: Was the exposure measured in a valid and reliable way?*

*Q4: Were objective, standard criteria used for measurement of the condition?*

*Q5: Were confounding factors identified?*

*Q6: Were strategies to deal with confounding factors stated?*

*Q7: Were the outcomes measured in a valid and reliable way?*

*Q8: Was appropriate statistical analysis used?*

**Supplementary Table 4**: Quality assessment using the Joanna Briggs Institute (JBI) Critical Appraisal Checklist for cohort, case-control, and mixed-method studies.

| **Author** | **Type** | **Q1** | **Q2** | **Q3** | **Q4** | **Q5** | **Q6** | **Q7** | **Q8** | **Q9** | **Q10** | **Q11** | **Total** | **Quality** |
| --- | --- | --- | --- | --- | --- | --- | --- | --- | --- | --- | --- | --- | --- | --- |
| Shelby | Cohort | Y | Y | Y | Y | Y | Y | Y | Y | Y | NA | Y | 100% | High |
| Elliott | Cohort | Y | Y | Y | Y | Y | Y | Y | Y | Y | NA | Y | 100% | High |
| McGinty | Cohort | Y | Y | Y | Y | Y | Y | Y | Y | Y | NA | Y | 100% | High |
| Porter | Case-Control | Y | U | Y | Y | Y | Y | Y | Y | Y | Y | - | 90% | High |
| Soriano | Mixed | Y | Y | Y | Y | Y | Y | Y | Y | Y | NA | Y | 100% | High |

*Q1: Were the two groups similar and recruited from the same population?*

*Q2: Were the exposures measured similarly to assign people to both exposed and unexposed groups?*

*Q3: Was the exposure measured in a valid and reliable way?*

*Q4: Were confounding factors identified?*

*Q5: Were strategies to deal with confounding factors stated?*

*Q6: Were the groups/participants free of the outcome at the start of the study (or at the moment of exposure)?*

*Q7: Were the outcomes measured in a valid and reliable way?*

*Q8: Was the follow up time reported and sufficient to be long enough for outcomes to occur?*

*Q9: Were participants analyzed in the groups to which they were randomized?*

*Q10: Were strategies to address incomplete follow up utilized?*

*Q11: Was appropriate statistical analysis used?*

**Supplementary Table 5**: Quality assessment using the Joanna Briggs Institute (JBI) Critical Appraisal Checklist for qualitative and mixed-method studies.

| **Author** | **Type** | **Q1** | **Q2** | **Q3** | **Q4** | **Q5** | **Q6** | **Q7** | **Q8** | **Q9** | **Q10** | **Total** | **Quality** |
| --- | --- | --- | --- | --- | --- | --- | --- | --- | --- | --- | --- | --- | --- |
| Harrison | Qualitative | Y | Y | Y | Y | Y | Y | N | Y | Y | Y | 90% | High |
| Regan Sterba | Qualitative | Y | Y | Y | Y | Y | N | N | Y | Y | Y | 80% | High |
| Brandzel | Qualitative | Y | Y | Y | Y | Y | Y | N | Y | Y | Y | 90% | High |
| Koo | Mixed | Y | Y | Y | Y | Y | N | N | Y | Y | Y | 80% | High |

*Q1: Is there congruity between the stated philosophical perspective and the research methodology?*

*Q2: Is there congruity between the research methodology and the research question or objectives?*

*Q3: Is there congruity between the research methodology and the methods used to collect data?*

*Q4: Is there congruity between the research methodology and the representation and analysis of data?*

*Q5: Is there congruity between the research methodology and the interpretation of results?*

*Q6: Is there a statement locating the researcher culturally or theoretically?*

*Q7: Is the influence of the researcher on the research, and vice-versa, addressed?*

*Q8: Are participants, and their voices, adequately represented?*

*Q9: Is the research ethical according to current criteria or, for recent studies, and is there evidence of ethical approval by an appropriate body?*

*Q10: Do the conclusions drawn in the research report flow from the analysis, or interpretation, of the data?*

**Supplementary Table 6: PRISMA 2020 Checklist**

| **Section and Topic** | **Item #** | **Checklist item** | **Location where item is reported** |
| --- | --- | --- | --- |
| **TITLE** | | |  |
| Title | 1 | Identify the report as a systematic review. | Title |
| **ABSTRACT** | | |  |
| Abstract | 2 | See the PRISMA 2020 for Abstracts checklist. | See Checklist |
| **INTRODUCTION** | | |  |
| Rationale | 3 | Describe the rationale for the review in the context of existing knowledge. | Intro p3 |
| Objectives | 4 | Provide an explicit statement of the objective(s) or question(s) the review addresses. | Intro p3 |
| **METHODS** | | |  |
| Eligibility criteria | 5 | Specify the inclusion and exclusion criteria for the review and how studies were grouped for the syntheses. | Methods p4 |
| Information sources | 6 | Specify all databases, registers, websites, organisations, reference lists and other sources searched or consulted to identify studies. Specify the date when each source was last searched or consulted. | Methods p3 |
| Search strategy | 7 | Present the full search strategies for all databases, registers and websites, including any filters and limits used. | Supplementary Table 1 |
| Selection process | 8 | Specify the methods used to decide whether a study met the inclusion criteria of the review, including how many reviewers screened each record and each report retrieved, whether they worked independently, and if applicable, details of automation tools used in the process. | Methods p4 |
| Data collection process | 9 | Specify the methods used to collect data from reports, including how many reviewers collected data from each report, whether they worked independently, any processes for obtaining or confirming data from study investigators, and if applicable, details of automation tools used in the process. | Methods p4 |
| Data items | 10a | List and define all outcomes for which data were sought. Specify whether all results that were compatible with each outcome domain in each study were sought (e.g. for all measures, time points, analyses), and if not, the methods used to decide which results to collect. | Methods p4 |
|  | 10b | List and define all other variables for which data were sought (e.g. participant and intervention characteristics, funding sources). Describe any assumptions made about any missing or unclear information. | Methods p4 |
| Study risk of bias assessment | 11 | Specify the methods used to assess risk of bias in the included studies, including details of the tool(s) used, how many reviewers assessed each study and whether they worked independently, and if applicable, details of automation tools used in the process. | Methods p4 |
| Effect measures | 12 | Specify for each outcome the effect measure(s) (e.g. risk ratio, mean difference) used in the synthesis or presentation of results. | Methods p4 |
| Synthesis methods | 13a | Describe the processes used to decide which studies were eligible for each synthesis (e.g. tabulating the study intervention characteristics and comparing against the planned groups for each synthesis (item #5)). | Methods p4 |
|  | 13b | Describe any methods required to prepare the data for presentation or synthesis, such as handling of missing summary statistics, or data conversions. | Methods p4 |
|  | 13c | Describe any methods used to tabulate or visually display results of individual studies and syntheses. | Methods p4 |
|  | 13d | Describe any methods used to synthesize results and provide a rationale for the choice(s). If meta-analysis was performed, describe the model(s), method(s) to identify the presence and extent of statistical heterogeneity, and software package(s) used. | Methods p4 |
|  | 13e | Describe any methods used to explore possible causes of heterogeneity among study results (e.g. subgroup analysis, meta-regression). | Methods p4 |
|  | 13f | Describe any sensitivity analyses conducted to assess robustness of the synthesized results. | Methods p4 |
| Reporting bias assessment | 14 | Describe any methods used to assess risk of bias due to missing results in a synthesis (arising from reporting biases). | Methods p4 |
| Certainty assessment | 15 | Describe any methods used to assess certainty (or confidence) in the body of evidence for an outcome. | Methods p4 |
| **RESULTS** | | |  |
| Study selection | 16a | Describe the results of the search and selection process, from the number of records identified in the search to the number of studies included in the review, ideally using a flow diagram. | Results p4-5; Figure 1 |
|  | 16b | Cite studies that might appear to meet the inclusion criteria, but which were excluded, and explain why they were excluded. | Figure 1 |
| Study characteristics | 17 | Cite each included study and present its characteristics. | Table 1 |
| Risk of bias in studies | 18 | Present assessments of risk of bias for each included study. | Results p5; Supplementary tables 2-5 |
| Results of individual studies | 19 | For all outcomes, present, for each study: (a) summary statistics for each group (where appropriate) and (b) an effect estimate and its precision (e.g. confidence/credible interval), ideally using structured tables or plots. | Tables 2-3 |
| Results of syntheses | 20a | For each synthesis, briefly summarise the characteristics and risk of bias among contributing studies. | Results 4-8 |
|  | 20b | Present results of all statistical syntheses conducted. If meta-analysis was done, present for each the summary estimate and its precision (e.g. confidence/credible interval) and measures of statistical heterogeneity. If comparing groups, describe the direction of the effect. | Results p4-8 |
|  | 20c | Present results of all investigations of possible causes of heterogeneity among study results. | Results p4-8 |
|  | 20d | Present results of all sensitivity analyses conducted to assess the robustness of the synthesized results. | Results p4-8 |
| Reporting biases | 21 | Present assessments of risk of bias due to missing results (arising from reporting biases) for each synthesis assessed. | Results p4-8 |
| Certainty of evidence | 22 | Present assessments of certainty (or confidence) in the body of evidence for each outcome assessed. | Results p4-8 |
| **DISCUSSION** | | |  |
| Discussion | 23a | Provide a general interpretation of the results in the context of other evidence. | Discussion p8-10 |
|  | 23b | Discuss any limitations of the evidence included in the review. | Discussion p9-10 |
|  | 23c | Discuss any limitations of the review processes used. | Discussion p10 |
|  | 23d | Discuss implications of the results for practice, policy, and future research. | Discussion p8-10 |
| **OTHER INFORMATION** | | |  |
| Registration and protocol | 24a | Provide registration information for the review, including register name and registration number, or state that the review was not registered. | Title Page |
|  | 24b | Indicate where the review protocol can be accessed, or state that a protocol was not prepared. | Title Page |
|  | 24c | Describe and explain any amendments to information provided at registration or in the protocol. | Title Page |
| Support | 25 | Describe sources of financial or non-financial support for the review, and the role of the funders or sponsors in the review. | Title Page |
| Competing interests | 26 | Declare any competing interests of review authors. | Title Page |
| Availability of data, code and other materials | 27 | Report which of the following are publicly available and where they can be found: template data collection forms; data extracted from included studies; data used for all analyses; analytic code; any other materials used in the review. | Methods p3-4 |
